# Between-sexes differences in lumbopelvic muscle mechanical properties of non-climacteric adults: a cross-sectional design

**DOI:** 10.1038/s41598-023-48984-8

**Published:** 2023-12-07

**Authors:** Daiana Priscila Rodrigues-de-Souza, Azahara Casas-Castro, María Cristina Carmona-Pérez, Lourdes García-Luque, Sandra Alcaraz-Clariana, Juan Luis Garrido-Castro, Francisco Alburquerque-Sendín

**Affiliations:** 1 Department of Nursing, Pharmacology and Physical Therapy, Faculty of Medicine and Nursing, University of Córdoba, 14004 Córdoba, Spain; 2 Maimonides Biomedical Research Institute of Cordoba (IMIBIC), 14004 Córdoba, Spain; 3 Department of Computer Science and Numerical Analysis, Rabanales Campus, University of Córdoba, 14071 Córdoba, Spain

**Keywords:** Physiology, Signs and symptoms, Urology

## Abstract

The lumbopelvic muscle mechanical properties (MMPs) are clinically relevant, but their dependence on sex remains unknown. Therefore, this study aimed to identify if lumbopelvic MMPs depend on the sex in a young adult population. Thirty-five healthy nulliparous women and 35 healthy men were analyzed (age range: 18–50). Lumbopelvic MMPs, that is, tone, stiffness, elasticity, relaxation and creep, assessed with MyotonPRO®, and pelvic floor (PF) health questionnaires were compared between-sexes. Intra-group correlations between sociodemographic and clinical data, and MMPs were also determined. The MMPs of PF were different between healthy non-climacteric adults of both sexes, with women showing higher values of tone and stiffness and lower values of elasticity and viscoelastic properties than men (in all cases, *p* < 0.03). At lumbar level, tone and stiffness were higher for men at both sides (in all cases, *p* < 0.04), and relaxation was lower at left side (*p* = 0.02). The MMPs showed few correlations with sociodemographic data within women. However, within males, there were positive correlations for PF stiffness and viscoelastic parameters with age, BMI and function (0.334 < r < 0.591) and, at lumbar level, negative correlations for tone and stiffness ( − 0.385 < r < −0.590) and positive correlations for viscoelastic properties (0.564 < r < 0.719), with BMI. This indicated that between-sexes differences of lumbopelvic MMPs depend on the specific location of assessment in healthy non-obese young individuals. Women show higher tone and stiffness and lower elasticity and viscoelasticity than men, at PF level.

## Introduction

According to the regional interdependence concept^1,2^, the lumbopelvic region is a functional unit^3^ that could have specific characteristics depending on sex^4,5^. The pelvic floor (PF) includes a complex anatomic structure, with fascial components (endopelvic fascia, ligaments, or perineal membrane), muscular components (pelvic floor muscles (PFM) including levator ani muscles, muscles of the urogenital diaphragm, and superficial perineal muscles)^6^, and neural components (both afferent and efferent nerves, visceral and somatic)^7^. Despite the skeletal and visceral differences, the histomorphology^8^ and architecture^9^ of PFM are similar between female and male individuals. The most relevant anatomic difference is the genital hiatus, which is broader in women^10,11^. Furthermore, the PFM are considered an integral part of the trunk and lumbopelvic stability^12,13^. According to this, in women populations, it has been shown that pelvic floor disorders (PFD) can cause pain, disability, and instability of the spine^14^, and lumbopelvic interventions can improve PFD, such as stress urinary incontinence^15^. In men, chronic pelvic pain syndrome is associated with spasms in the quadratus lumborum and the iliopsoas and hypomobility of the thoracolumbar spine^16^. However, other data, such as the lumbopelvic muscle mechanical properties (MMPs), remain unknown, even when their status is relevant in both sexes^17,18^. The study of MMPs is of interest even in asymptomatic populations, a less studied population^19,20^, since the consequences of risk factors for PFD on the PFM, such as an alteration of MMPs, may not be evident until later in life^21^.

Usually, the PFM analysis focuses on strength, assessed with dynamometry^22^, and muscle activity, assessed with electromyography^23^, both in women and men populations^24^. However, these methodologies do not adequately measure muscle tone or other MMPs, as they do not directly measure resistance to change in muscle length^18^. Moreover, there is neither a single accepted standardized way of measuring muscle tone nor normative values^25^, and the palpation method is not entirely validated^26^. Therefore, standardizing a reliable and reproducible examination is needed^27^. In this line, new technologies allow the evaluate MMPs, as is the case of MyotonPRO (Myoton AS, Tallinn, Estonia), a manual myotonometric device that does not require high levels of expertise^28^, contrary to other assessment tools, such as ultrasound imaging, and can be used by novices with acceptable results^29^, following standardized protocols^30^. Although manual myotonometry has been successfully applied to different clinical states and locations, including lumbar^31,32^ and PF^18,28^ regions, no study has determined whether the lumbopelvic MMPs are different between sexes.

Thus, this study aimed to identify if lumbopelvic MMPs depend on sex in a young adult population. Secondarily, intra-sex associations between lumbopelvic MMPs and sociodemographic characteristics were analyzed.

## Methods

### Design

A cross-sectional case–control study with consecutive case recruitment was designed. The strengthening the reporting of observational studies in epidemiology (STROBE) method was used. The Research Ethics Committee of Córdoba (registration number 5174, October 2021) approved the project. The study was conducted in accordance with the Declaration of Helsinki. All participants signed the informed consent form.

### Participants

The inclusion criteria for women were: healthy adult women, between 18 and 50 years old; nulliparous; without any type of delivery or pregnancy. The men group inclusion criterion was: healthy adult men, between 18 and 50 years old. Exclusion criteria for both sexes were: history of surgery at lumbopelvic level; engagement in regular physical training (only recreationally active, without high impact exercises, less than 6 h per week, was allowed); moderate or heavy occupational physical activity^33^; experience of any spinal pain in the six months before enrolment in the study^34^; climacteric period; any type of incontinence; body mass index (BMI) > 30 kg/m^2^. Participants of both sexes were paired by age (± 3 years).

### Sample size

To identify a moderate effect size (Cohen d index = 0.7) based on a minimum detectable change (MDC) of 0.86 and a pooled standard deviation of 1.23 for frequency (muscle tone), as previously reported^28^, an error type I of 0.05 and 0.80 of power, at least 34 women/men per group were necessary (G*power 3.1.9.2, t-test for difference between two independent measures).

### Procedures

As recommended, the patient emptied the bladder before the evaluation^35^. A physical therapist with more than five years of experience assessed the inclusion and exclusion criteria. Two validated questionnaires for women's PF assessment were applied. The Pelvic floor distress inventory (PFDI-20) includes 20 questions divided into three scales according to the symptoms: symptoms of genital prolapse, questions 1 to 6 (POPDI-6); colorectal-anal symptoms, questions 7 to 14 (CRADI-8); and urinary symptoms, questions 15 to 20 (UDI-6). The total score is the sum of the three blocks with a maximum score of 300, where higher scores mean high distress^36,37^. The Pelvic floor impact questionnaire (PFIQ-7) includes seven questions about the impact of symptoms on activities, relationships, or feelings concerning urinary prolapse (UIQ-7), colorectal-anal conditions (CRAIQ-7), and genital conditions (POPIQ-7). Again, the total score is the sum of the three blocks with a maximum score of 300, where a higher score means a high impact^36,37^. The International Prostate Symptoms Score (IPSS), which assesses the intensity of lower urinary tract symptoms during the past 30 days, was applied to the men^38,39^. It consists of 7 questions on a Likert scale with five possible answers. The final score was interpreted as follows: mild (0–7 points), moderate (8–19 points), and severe (20–35 points)^40^. Sociodemographic and clinical data, such as age, BMI, and level of physical activity according to the Global Physical Activity Questionnaire (GPAQ)^41^, were also collected.

**Figure 1:**
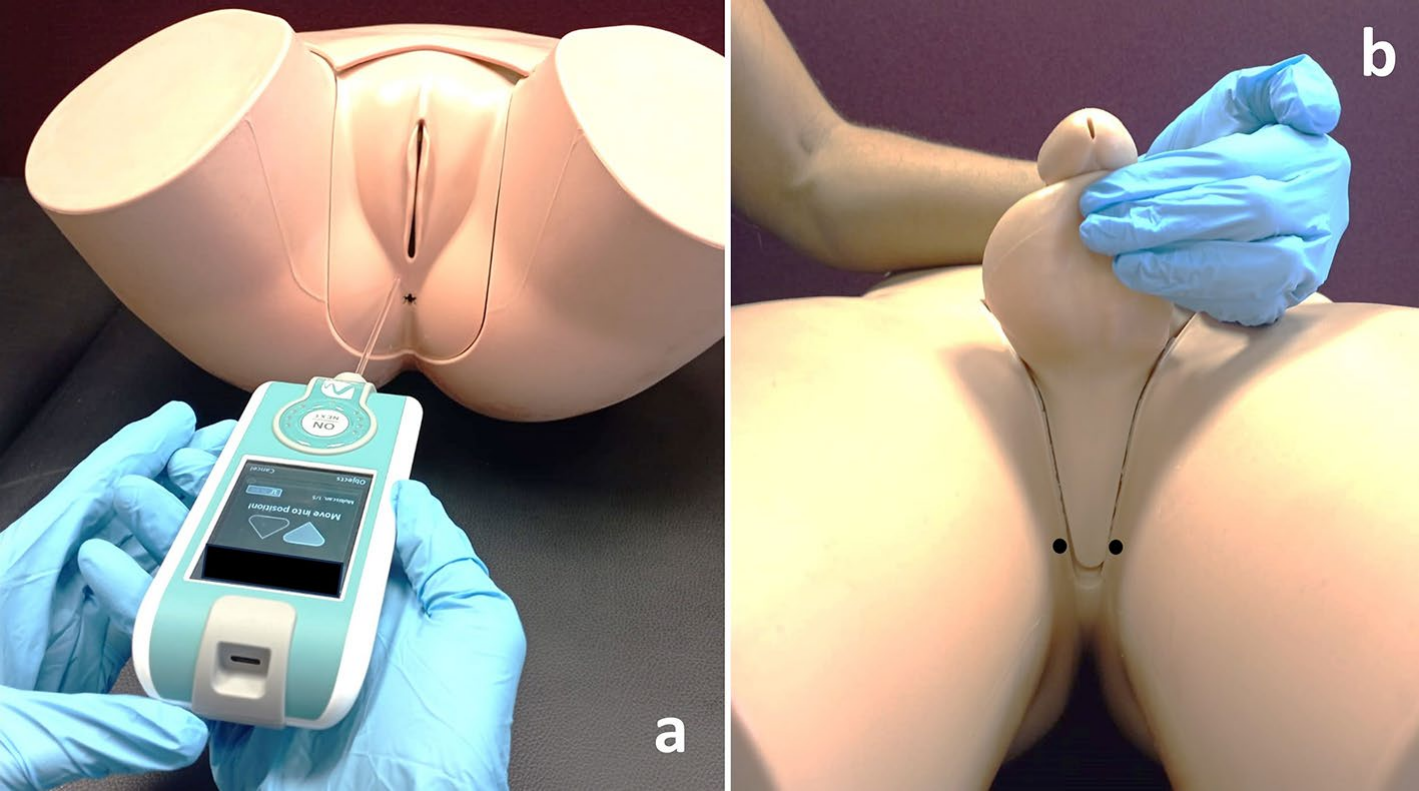
Assessment of the MMPs with MyotonPRO® device at PF level (anatomical models). (**a**) Woman evaluation with the 100 mm long probe (right side); (**b**) Anatomic location for the assessments in man (black dots).

The MMPs measurement protocol of lumbopelvic muscles is described elsewhere^28,42,43^. A physical therapist, previously trained in myotonometric measures (2 h of training in several body locations), carried out the protocol with a manual tonometer (MyotonPRO® Myoton AS, Tallinn, Estonia). The measurements were taken with the participants in supine position, with the knees flexed and the soles of the feet on the table, to ensure that both lower limbs were symmetrical and relaxed during the measurement. The measurement site was located by visualization and palpation in the largest area of muscle bulk during contraction (verbal order: “stop the flow of urine^44^) on both sides of the central perineal body. This area was selected because it contains the most contractile portion of the perineal muscles. Once the muscle was relaxed, a mark was placed with a dermographic marker to ensure the measurement site^18^. At this moment, the men were asked to keep the sexual organs away from the perineal body, without tension or traction of the skin^42^. The 100 mm long probe of the MyotonPRO® was placed perpendicular to the skin's surface, on the mark location, to perform the measurements (Fig. 1).

Lumbar MMPs were assessed with the patient in prone position for 2 min. The therapist identified the measurement points according to the visual and palpatory approach, at 2.5 cm to the right and left of L5 erector spinae to evaluate the erector spinae. In this case, the 35 mm probe was placed vertically on the skin surface of the muscle belly to perform the test^32^.

All recordings were performed during five seconds of apnea after exhalation^32^. The average of three mechanical impulses were applied for each assessment, according to the multiple mode of the MyotonPRO®. The coefficient of variation among the mechanical impulses was lower than 3% for the MMPs^28^. A randomization process was performed to establish the order of the evaluations (right/left), both in PF and lumbar muscles.

The description of the MMPs can be summarized as^45^: Frequency, characterizing muscle tension or tone in resting state (Hz); biomechanical properties, such as Stiffness (N/m) and logarithmic Decrement in the amplitude of oscillation (Ø), which defines the inverse of the elasticity; and viscoelastic properties, such as Relaxation time of stress (ms), and Creep (Deborah Number -De-), that characterizes fluidity. The MMPs evaluation has been demonstrated to be valid and reliable in different body locations^30^, including lumbar (intraclass correlation coefficient: intra-rater, from 0.80 to 0.99^43,46,47^) and PF (intraclass correlation coefficient: intra-rater, from 0.63 to 0.86; inter-rater, from 0.70 to 0.92, except for creep, that was from 0.40 to 0.46^18,28^) regions, in healthy individuals.

At the end of the procedures, a Visual analogue scale (VAS) was applied to identify any presence of pain and its intensity during the evaluation^28^. All procedures lasted less than 30 min for each subject.

### Statistical analysis

Frequencies and percentages were used to describe qualitative data. Age, BMI and all outcomes showed a normal distribution of data (Kolmogorov–Smirnov test *p* > 0.05) and were described by the mean, standard deviation or 95% confidence interval (95%CI), and range. Questionnaire variables were not normally distributed and were described by median and interquartile range. Since some between-sides statistical differences were identified (*p* < 0.05) for the MMPs when submitted to paired Student t-tests, each side was analyzed separately.

Sociodemographic and clinical data were compared between sexes with unrepeated Student t-tests, except for the level of physical activity, which was compared with X^2^ test. For the primary aim of the study, unpaired Student t-tests were applied to identify differences in MMPs of PF and lumbar muscles between both sexes. To identify intra-group associations between MMPs and sociodemographic and clinical features in each group, Pearson r or Spearman ρ (r_s_) coefficients were calculated. Correlations were considered negligible (0.0 to 0.19), fair (0.20 to 0.39), moderate (0.40 to 0.69), strong (0.70 to 0.89), or almost perfect (0.0 to 1.00)^[﻿48^.

All contrasts were bilateral, and *p* < 0.05 was considered significant. The IBM-SPSS version 28® software (Chicago, IL, USA) was used for the analyses.

## Results

### Sociodemographic and clinical data

Thirty-five nulliparous women and 35 men were analyzed. There were no differences between groups in age, while BMI was higher in men (mean difference: −2.07 kg/m^2^, 95%CI = −3.78, −0.36). Other sociodemographic and clinical data of both groups are included in Table 1. No individual reported pain (VAS = 0) due to the evaluation.

**Table 1:** Sociodemographic and clinical data of the sample.

	Women group (*n* = 35)	Men group (*n* = 35)	P-value
Age (years)	25.17 ± 6.67 [19–46]	25.09 ± 9.1 [19–45]	0.965
BMI (Kg/m^2^)	22.32 ± 2.91 [18.10–28.20]	24.40 ± 4.17 [19.25–29.91]	0.019*
PFDI-20	5.75 (4.17)		
PFIQ-7	1.93 (3.98)		
IPSS		2.19 (3)	
GPAQ (low/moderate/high levels)	1/21/13	2/23/10	0.665

Values expressed as frequencies, means ± SD [range], or median (interquartile range). *Significant difference (*P* < 0.05) between groups. Abbreviations: BMI: body mass index; PFDI: Pelvic Floor Distress Inventory; PFIQ: Pelvic Floor Impact Questionnaire; IPSS: International Prostate Symptoms Score; GPAQ: Global Physical Activity Questionnaire.

### Between sexes differences in lumbopelvic MMPs

All MMPs of PF were different between sexes at both sides, with women showing higher values of tone and stiffness, and lower elasticity and viscoelasticity (in all cases, p < 0.03). At lumbar level, tone and stiffness were higher for men on both sides, and relaxation was lower on the left side. No differences were identified for decrement and creep (Table 2).

**Table 2:** MMPs of PF and lumbar muscles of both sides and sexes.

		Side	Women group (*n* = 35)	Men group (*n* = 35)	Between-sexes difference	p-value
PF	Frequency (Hz)	Right	14.97 ± 2.09	12.19 ± 0.98	2.78 (2.01 ; 3.56)	< 0.001*
		Left	15.39 ± 2.08	12.55 ± 1.43	2.84 (1.99 ; 3.69)	< 0.001*
	Stiffness (N/m)	Right	223.203 ± 68.11	143.63 ± 21.83	79.57 (55.45 ; 103.70)	< 0.001*
		Left	231.23 ± 61.18	151.11 ± 26.81	80.11 (57.58 ; 102.65)	< 0.001*
	Decrement	Right	1.04 ± 0.22	0.92 ± 0.22	0.12 (0.01 ; 0.22)	0.029*
		Left	1.05 ± 0.21	0.93 ± 0.22	0.13 (0.02 ; 0.23)	0.016*
	Relaxation (ms)	Right	18.05 ± 2.71	23.45 ± 3.85	-5.40 (-6.99 ; -3.81)	< 0.001*
		Left	17.41 ± 2.52	22.98 ± 3.66	-5.58 (-7.07 ; -4.08)	< 0.001*
	Creep (De)	Right	0.96 ± 0.10	1.16 ± 0.31	-0.20 (-0.31 ; -0.09)	< 0.001*
		Left	0.94 ± 0.96	1.15 ± 0.26	-0.22 (-0.31 ; -0.12)	< 0.001*
Lumbar	Frequency (Hz)	Right	13.40 ± 1.53	14.22 ± 1.69	-0.82 (-1.59 ; -0.05)	0.036*
		Left	13.24 ± 1.59	14.46 ± 1.56	-1.22 (-1.97 ; -0.47)	0.002*
	Stiffness (N/m)	Right	212.83 ± 54.00	244.71 ± 49.22	-31.89 (-56.53 ; -7.24)	0.012*
		Left	205.69 ± 54.56	243.40 ± 52.31	-37.71 (-63.21 ; -12.22)	0.004*
	Decrement	Right	1.07 ± 0.23	1.11 ± 0.28	-0.04 (-0.17 ; 0.08)	0.485
		Left	1.03 ± 0.27	1.09 ± 0.42	-0.06 (-0.23 ; 0.11)	0.493
	Relaxation (ms)	Right	22.67 ± 4.21	21.15 ± 4.60	1.52 (-0.58 ; 3.63)	0.153
		Left	23.04 ± 4.23	20.58 ± 4.17	2.46 (0.46 ; 4.47)	0.017*
	Creep (De)	Right	1.30 ± 0.26	1.27 ± 0.27	0.04 (-0.09 ; 0.16)	0.582
		Left	1.31 ± 0.25	1.22 ± 0.26	0.09 (-0.03 ; 0.21)	0.137

Values expressed as means ± SD and between means difference (95%CI). *Significant difference (P < 0.05) between groups. Abbreviations: PF: pelvic floor.

### Correlations between sociodemographic and clinical status, and lumbopelvic MMPs depending on the sex

For the women group, age showed no correlation with any lumbopelvic MMP. However, at PF level, the BMI was directly related to the viscoelastic properties at both sides in fair to moderate fashion (0.364 < r < 0.452), that is, the higher BMI, the higher relaxation and creep. Tone, stiffness and decrement of PF showed no correlations with BMI.

At lumbar level, the women showed a consistent trend of correlations between BMI and MMPs. Thus, tone and stiffness were moderate and inversely related to BMI (-0.412 < r < -0.480), while viscoelastic properties were moderate and directly related to BMI (0.465 < r < 0.522). The lumbar elasticity showed no relation with BMI. Finally, considering the clinical status of PF, no correlation was identified between PFIQ-7 and PFDI-20 and any lumbopelvic MMP (Table 3).

**Table 3:** Bivariate correlations between lumbopelvic MMPs and age, BMI, PFDI-20 and PFIQ-7 of women group (n = 35).

		Side	Age	BMI	PFDI-20	PFIQ-7
PF	Frequency	Right	0.294 ; 0.086	−0.319 ; 0.056	−0.025 ; 0.888	0.186 ; 0.300
		Left	0.147 ; 0.399	−0.284 ; 0.098	−0.086 ; 0.622	0.154 ; 0.392
	Stiffness	Right	0.232 ; 0.179	−0.329 ; 0.053	−0.120 ; 0.493	0.152 ; 0.398
		Left	0.165 ; 0.343	−0.224 ; 0.196	−0.059 ; 0.736	0.108 ; 0.551
	Decrement	Right	0.237 ; 0.170	−0.161 ; 0.354	0.138 ; 0.430	0.216 ; 0.227
		Left	0.295 ; 0.086	−0.013 ; 0.941	0.134 ; 0.442	0.306 ; 0.084
	Relaxation	Right	−0.241 ; 0.163	**0.452 ; 0.006**	0.168 ; 0.336	−0.253 ; 0.155
		Left	−0.188 ; 0.279	**0.368 ; 0.030**	0.089 ; 0.611	−0.147 ; 0.415
	Creep	Right	−0.148 ; 0.395	**0.391 ; 0.020**	0.232 ; 0.180	−0.236 ; 0.186
		Left	−0.139 ; 0.425	**0.364 ; 0.032**	0.135 ; 0.441	−0.022 ; 0.903
Lumbar	Frequency	Right	0.082 ; 0.638	**−0.480 ; 0.004**	−0.019 ; 0.915	−0.098 ; 0.587
		Left	0.055 ; 0.755	**−0.449 ; 0.007**	0.004 ; 0.982	−0.112 ; 0.535
	Stiffness	Right	0.272 ; 0.115	**−0.480 ; 0.004**	−0.079 ; 0.652	0.071 ; 0.696
		Left	0.258 ; 0.135	**−0.412 ; 0.014**	−0.047 ; 0.789	−0.040 ; 0.825
	Decrement	Right	0.244; 0.166	0.187 ; 0.283	−0.020 ; 0.909	0.235 ; 0.188
		Left	0.273 ; 0.118	0.240 ; 0.171	−0.127 ; 0.474	0.037 ; 0.840
	Relaxation	Right	−0.060 ; 0.731	**0.522 ; 0.001**	0.050 ; 0.774	0.072 ; 0.689
		Left	0.015 ; 0.931	**0.510 ; 0.002**	0.070 ; 0.688	0.122 ; 0.498
	Creep	Right	0.044 ; 0.801	**0.473 ; 0.004**	0.042 ; 0.812	0.059 ; 0.746
		Left	0.116 ; 0.507	**0.465 ; 0.005**	0.051 ; 0.770	0.076 ; 0.676

Values expressed as Pearson r or Spearman ρ (r_s_) coefficients; p−value. Bold numbers mean significant correlation (p < 0.05). Abbreviations: BMI: body mass index; IPSS: International Prostate Symptoms Score; PF: pelvic floor.

For the men group, the age was correlated inversely with tone on the right side, and directly with decrement, relaxation and creep in fair to moderate intensity, at both sides of PF (|0.334|< r <|0.446|). The age was also directly related to the decrement at lumbar level (0.395 < r < 0.470). Regarding BMI, all lumbopelvic MMPs, except the PF tone, were related with the BMI in fair to strong intensity at both sides (|0.385|< r <|0.719|). In all cases, the higher BMI, the lower tone and stiffness, while the higher BMI, the higher decrement, relaxation and creep.

The IPSS questionnaire showed some correlations with MMPs only at PF level. Specifically, fair to moderate relations were identified between IPSS, and frequency on the right side and stiffness on the left side in an inverse fashion, and between IPSS, and decrement of the left side, relaxation of the right side and creep of both sides, in a direct fashion (|0.350 < r_s_ < 0.438|) (Table 4).

**Table 4:** Bivariate correlations between lumbopelvic MMPs and age, BMI and IPSS of men group (n = 35).

		Side	Age	BMI	IPSS
PF	Frequency	Right	**−0.417 ; 0.013**	−0.224 ; 0.195	**−0.359 ; 0.045**
		Left	−0.239 ; 0.167	−0.091 ; 0.603	0.090 ; 0.623
	Stiffness	Right	0.027 ; 0.876	**0.483 ; 0.003**	0.073 ; 0.689
		Left	0.015 ; 0.932	**0.446 ; 0.007**	**−0.357 ; 0.046**
	Decrement	Right	**0.334 ; 0.047**	**0.509 ; 0.002**	0.045 ; 0.809
		Left	**0.337 ; 0.048**	**0.502 ; 0.002**	**0.365 ; 0.040**
	Relaxation	Right	**0.446 ; 0.007**	**0.588 ; < 0.001**	**0.438 ; 0.012**
		Left	**0.382 ; 0.024**	**0.396 ; 0.018**	0.194 ; 0.287
	Creep	Right	**0.392 ; 0.020**	**0.680 ; < 0.001**	**0.431 ; 0.014**
		Left	**0.434 ; 0.009**	**0.591 ; < 0.001**	**0.350 ; 0.049**
Lumbar	Frequency	Right	0.057 ; 0.746	**−0.590 ; < 0.001**	0.006 ; 0.973
		Left	−0.015 ; 0.933	**−0.466 ; 0.005**	−0.142 ; 0.439
	Stiffness	Right	0.224 ; 0.197	**−0.408 ; 0.015**	0.106 ; 0.564
		Left	0.245 ; 0.156	**−0.385 ; 0.037**	−0.039 ; 0.834
	Decrement	Right	**0.470 ; 0.004**	**0.564 ; < 0.001**	0.224 ; 0.218
		Left	**0.395 ; 0.019**	**0.719 ; < 0.001**	0.215 ; 0.236
	Relaxation	Right	0.060 ; 0.732	**0.648 ; < 0.001**	0.143 ; 0.436
		Left	0.047 ; 0.787	**0.634 ; < 0.001**	0.189 ; 0.301
	Creep	Right	0.128 ; 0.463	**0.666 ; < 0.001**	0.173 ; 0.343
		Left	0.145 ; 0.407	**0.657 ; < 0.001**	0.209 ; 0.252

Values expressed as Pearson r or Spearman ρ (r_s_) coefficients; p-value. Bold numbers mean significant correlation (*p* < 0.05). Abbreviations: BMI: body mass index; IPSS: International Prostate Symptoms Score; PF: pelvic floor.

## Discussion

The results showed that all MMPs of PF are different between healthy non-climacteric adults of both sexes, with women showing higher values of tone and stiffness and lower values of elasticity and viscoelastic properties than men. On the contrary, tone, stiffness and relaxation on the left side of lumbar muscles were different between sexes, being men that had higher tone and stiffness and lower relaxation than women. Further, the existence of significant and non-significant correlations between lumbopelvic MMPs and sociodemographic and clinical data depended on each sex. Thus, women lumbopelvic MMPs were not correlated with age, while men MMPs on both locations showed consistent trends of correlations with age, with higher intensity and PF level. Similarly, men showed more and higher intensity correlations between BMI and lumbopelvic MMPs, than women, mainly when PF was considered. Regarding pelvic clinical values, only the MMPs of men at PF level showed some correlations, in this case, with the IPSS. In summary, lumbopelvic MMPs are different between sexes, and these differences depend on the location, with PF being more rigid and less viscoelastic in women and showing relations to age and clinical status in men.

No individual reported pain or any discomfort during the evaluation, which reinforces the idea that the determination of MMPs can be applied in clinical setting, due to its innocuousness.

### Location dependence of the between sexes differences of the lumbopelvic MMPs

The magnitude of the differences in MMPs of PF between sexes exceeds the MDC established for these measures^28^. Thus, although the genital hiatus is broader in women^10,11^ and is part of the main source of PF weakness, the more robust development of connective tissue in the female PF^9^ can explain the higher tone and stiffness and the lower elasticity and viscoelasticity of PF in women. In fact, the perineal body and deep perineal muscles are larger, and their bifurcation near the urethra is broader and longer in women than in men. Moreover, the complex of the deep perineal muscle and perineal body forms a perineal membrane in women, while, in men, deep perineal muscle is a relatively small, median structure^9^. Other between-sexes differences in PFM could also justify the need for higher rigidity of women's PF. For example, the male PF has a much steeper and narrower funnel shape than the female, which levator ani muscles develop a higher working load^9^. Furthermore, most men are not familiar with PFM contractions and training, probably due to the lower incidence of PFDs and, consequently, the lower necessity to perform specific exercises focused on the PFM^49^, which could lead to less tone at this level.

Several between-sex differences in MMPs were also identified at lumbar level, with men demonstrating higher tone and stiffness, but below the MDC for these muscles^47^. It is well known that men’s muscles can be stronger than women's ones^50^, which can also determine a higher stiffness in different contraction states in men^51^. All this can be explained by the higher prevalence of slower type-I and type-IIA fibers, which could show less tension and stiffness in females than males^52^, although, in the current study, the elasticity and viscoelasticity were similar during rest for both sexes. In summary, women's PF is more rigid and less viscoelastic than men's, while lumbar muscles show fewer differences between sexes.

The current results did not show a constant pattern of between-sides asymmetries. Thus, only lumbar relaxation was different depending on the side, but with fewer between-sides differences (< 1.0 ms) than between-sexes differences (> 1.5 ms). Nevertheless, it was reported that the symmetry of MMPs could depend on specific states and disorders. Thus, when asymmetrical processes are considered, such as scoliosis^53,54^, surgery of Achilles tendon rupture^55^ or obstetric scars and lesions^56,57^ associated to episiotomies^58^ and deliveries^42^, bilateral differences in MMPs are common. However, when no pathologic or traumatic processes occur, fewer asymmetries are found in PF^42^ and other regions^﻿59^, as shown by our sample of both sexes.

Regarding the correlation analysis, on the one hand, MMPs of PF showed no relationship with age and clinical status of PF and some low but significant associations with BMI in women. On the other hand, in men, MMPs were more correlated with age and BMI, and even the clinical status of PF showed correlations with MMPs of PF. This could mean that other features than age or BMI, such as hormonal status^60^ or exercise^34^, could be related to the MMPs of PF in nulliparous adult women. Moreover, the absence of relation between the MMPs and age found in women could also be due to the age range of our sample, exclusively composed of young adults. In fact, in healthy subjects, sarcopenia begins around the age of 50 years, which was the high range limit of the current sample^61^, and the lower muscular development of the female sex and the presence of adrenal androgens after menopause could make sarcopenia less apparent before the climacteric period^62^.

### Clinical relevance of the assessment of lumbopelvic MMPs in both sexes

The assessment of MMPs in the lumbopelvic ring is of clinical relevance, since PF hypertonicity is often associated with urological, gynecological, gastrointestinal and sexual problems as well as chronic pelvic pain in both sexes﻿^63^, and tone alterations of PFM are related to postoperative male urinary incontinence^64^. Similarly, stiffness, as the resistance to deformation, is also relevant for PFM assessment^65^ and should be measured quantitatively^25^. Thus, a fast, innocuous and reliable MMPs assessment of PFM can help in PF examination^27^. In this sense, the internal vaginal probes used for dynamometry may induce changes in PFM recruitment by the mere presence of the probes^22^, and the size of levator hiatus can condition the evaluations^66^ in women. In men, a digital rectal examination is often used to evaluate PFM, but this invasive technique might cause discomfort, resulting in a lack of cooperation and poor outcomes﻿^67^. On the contrary, the external application of myotonometry and its innocuousness is described as an advantage^68^.

Other previous methods used to objectively fvassess perineal body tone included, in men, the Beco perineometer, which measures the introflection values of the perineal body relative to the ischiatic spines. Nevertheless, this mechanical device determines the descent of the perineum in centimeters^64^, which could be inadequate for muscle assessment. Furthermore, the PFM assessment does not commonly consider bilateral evaluations^69,70^, as occurs with dynamometry and electromyography, while the determination of MMPs allows individualizing each side, which is considered relevant under certain conditions, such as asymmetric diseases or deliveries^6,25,42^ as previously commented. Moreover, the assessment of MMPs with manual tonometry has demonstrated relevance in different fields^71,72^, including lumbar ^32,43,46^ and PFM^18,28,73^.

In summary, the differences in MMPs depending on sex suggest that the physiology of these structures could also be different in healthy states, and, consequently, specific analysis approaches could be recommended for each sex. Moreover, the external application of myotonometry, its innocuousness, speed of application, bilateral evaluation, and low need for training^29^, increase the interest of this method in clinical setting.

## Limitations of the study

Some limitations should be recognized. Only healthy non-obese young adults who did not practice regular exercise were included, and all women were nulliparous and non-climacteric. Therefore, the external validity of the results is limited to populations with similar characteristics. All evaluations were performed in the same rest positions, which could increase the consistency of the results, but other positions and states could lead to determining different results^74^. The menstrual cycle phase was not controlled in the women group, which could be relevant in clinical evaluation^60^. Future research, considering other ages and clinical states, is recommended.

## Conclusions

Between-sexes differences in lumbopelvic MMPs depend on the specific location of the assessment in healthy non-obese young individuals, only recreationally active. Thus, the MMPs of PF differ between healthy non-climacteric adults of both sexes, with women showing higher values of tone and stiffness and lower values of elasticity and viscoelastic properties than men. In contrast, lumbar MMPs show less and lower differences. Only MMPs of men are related to age, BMI and clinical status of PF. Lumbopelvic MMPs evaluation with external tonometry could be relevant in clinical setting, due to its innocuousness and bilateral applicability.

## Data Availability

The datasets generated during the current study are available from the corresponding author on reasonable request. The data are not publicly available due to privacy of research participants.
